# Dupuytren's contracture: a retrospective database analysis to assess clinical management and costs in England

**DOI:** 10.1186/1471-2474-12-73

**Published:** 2011-04-12

**Authors:** Robert A Gerber, Richard Perry, Robin Thompson, Christopher Bainbridge

**Affiliations:** 1 Clinical Development and Medical Affairs, Pfizer Global Development Headquarters, New London, CT, USA; 2 Adelphi, Macclesfield, Cheshire, UK; 3 Pfizer Ltd, Walton Oaks, Tadworth, Surrey, UK; 4 Pulvertaft Hand Centre, Derby, Derbyshire, UK

## Abstract

**Background:**

Dupuytren's disease is a fibro-proliferative disorder affecting ~3-5% of the UK population. Current surgical treatments for Dupuytren's contracture (DC) include fasciectomy and fasciotomy. We assessed the clinical management of DC in England over a 5-year period; associated NHS costs were assessed for a 1-year period.

**Methods:**

Hospital Episode Statistics were extracted from April 2003 to March 2008 for patients with Palmar Fascial Fibromatosis (ICD10 = M720) and DC-related procedures. Variables included demographics, OPCS, patient status and physician specialty. To estimate 2010-2011 costs, HRG4 codes and the National Schedule of Tariff 2010-11-NHS Trusts were applied to the 2007-2008 period.

**Results:**

Over 5 years, 75,157 DC admissions were recorded; 64,506 were analyzed. Mean admissions per year were 12,901 and stable. Day cases increased from 42% (2003-2004) to 62% (2007-2008). The percent of patients having two or more admissions per year increased from 5.5% in 2003-2004 to 26.1% in 2007-2008. Between 2003 and 2007, 91% of procedures were Fasciectomy. Revision of Fasciectomy and Fasciotomy each accounted for ~4%; Amputation for 1%. In 2007, classification was extended to identify Digital Fasciectomy, its Revision and Dermofasciectomy. In 2007-2008, admissions were: 70% Palmar Fasciectomy, 16% Digital Fasciectomy, 1.3% Other Fasciectomy, 4.4% Revision of Palmar Fasciectomy, 1.3% Revision of Digital Fasciectomy, 3.8% Division of Palmar Fascia, 2.6% Dermofasciectomy and 1.1% Amputation. 79% of cases were overseen by trauma and orthopaedic surgeons, 19% by plastic surgeons. Mean (±SD) inpatient hospital length of stay was 1.5 (±1.4) days in 2003-2004 and 1.0 (±1.3) days in 2007-2008. Total estimated costs for 1 year (2010-2011) were £41,576,141. Per-patient costs were £2,885 (day case) and £3,534 (inpatient). Costs ranged from £2,736 (day-case Fasciectomy) to £9,210 (day-case Revision Digital).

**Conclusions:**

Between 2003 and 2008, fasciectomy was the most common surgical procedure for DC in England. While procedure rates and physician specialties varied little, there was a reversal in surgical venue: inpatient operations decreased as day-case procedures increased. The change is likely due to economic trends and changes to the healthcare system. Estimated costs for 2010-2011 varied by procedure type and patient status. These findings can be used to understand clinical management of DC and guide healthcare policy.

## Background

Dupuytren's disease is a fibro-proliferative disorder affecting the palmar fascia whereby early nodular tissue becomes acellular, and a thick collagen cord develops. As the cord contracts, flexion deformity of the affected metacarpophalangeal or proximal interphalangeal joint ensues. Joint contracture is a common presenting complaint, as it can impair hand function at home, in the workplace and in social interactions [1]. There is no cure for Dupuytren's disease; however, treatment goals include removing or releasing the fibrotic cord to allow extension of the affected finger(s) and restoration of hand function. Common surgical approaches include limited fasciectomy (aponeurectomy), fasciotomy (aponeurotomy), percutaneous needle aponeurotomy and dermofasciectomy. The type of approach used depends on many factors, including but not limited to patient age, comorbid conditions and severity of disease.

While it is generally assumed that the majority of patients with Dupuytren's disease are native to or descendents of ancestors from northern Europe, epidemiologic studies have been conducted in numerous countries. Although published prevalence estimates vary widely, most studies have shown that prevalence increases with advancing age [2,3].

To our knowledge, the only published study to assess hospital costs associated with Dupuytren's disease was conducted by Maravic and Landais in France in 2005. In this study, we evaluated the clinical management of Dupuytren's disease over a 5-year period in England using data extracted from a National Health Service (NHS) database. Variables of interest included patient demographics, the types and frequencies of surgical procedures, day-case or inpatient admission status, and the specialty of the treating physician. Associated costs were estimated for a 1-year period. The aim of the study was to describe surgical treatment patterns and the associated costs for Dupuytren's contracture in England.

## Methods

Hospital Episode Statistics (HES) were extracted for 5 complete years between 2003 and 2008 (April-March periods) for subjects with a diagnosis code for Palmar Fascial Fibromatosis (International Classification of Diseases [ICD]-10 = M720). The database, provided by Caspe Healthcare Knowledge Systems (CHKS), contains details about admissions to NHS hospitals in England, including acute hospitals, primary care trusts and mental health trusts. It includes private patients treated in NHS hospitals, and patients who reside outside of England but receive care from treatment centres funded by the NHS. The database excludes patients treated in the private sector where the treatment is not funded by the NHS.

HES-based patient records contain demographic (eg, age, gender, ethnicity, residence, treatment location), clinical (eg, diagnoses, procedure codes), and administrative information (eg, dates of admission/discharge, time waited). For this analysis, information on subject demographics, Office of Population Census and Survey (OPCS)-4 codes (surgical procedure codes), day-case and hospital admissions, and treating physician specialty were collected. For each year, resource utilization was determined for all patients with an admission for Dupuytren's contracture, and the number of hospitalizations, length of hospital stay for Dupuytren's contracture, and type of admission (day case versus inpatient) were recorded. For this analysis, a hospital admission was defined as any case where a patient is admitted to a hospital as either a day case (a visit that excludes an overnight stay) or inpatient (≥1 night stay in the hospital). Elective procedures were defined as planned/scheduled; non-elective procedures were defined as unplanned/emergency.

Payment by results, introduced across the NHS in England and Wales in 2005, uses healthcare resource groups (HRGs) as a measure of care, based on both the diagnosis and the complexity of treatment. Their purpose is to group together similar clinical treatments that should cost an equivalent amount to deliver. This system aims primarily to provide a structure of a national fixed tariff, with a secondary aim to improve productivity and increase capacity throughout the NHS [4]. An individual tariff, which is based on a reference cost (ie, the weighted average costs for all English hospitals, is related to actual costs of a procedure. At least one code is assigned to each hospital episode to be funded by the patient's primary care trust [5]. The HRG4 codes are derived from a combination of diagnostic (ICD-10) and procedure (OPCS-4) codes derived from the patient's records [6]. To estimate total and per-patient costs, the 2010-2011 NHS National Schedule of Tariff [7] was mapped to the 2007-2008 procedure HRG4 treatment groupings to value all admissions. Per-patient costs were calculated because they provide the best estimate of a complicated case mix of multiple HRG codes and different frequencies of day-case and inpatient admissions. Costs associated with ancillary services such as follow-up visits and physiotherapy were not included.

All results are reported descriptively. Categorical data are presented as counts (n) and proportions (%). Continuous data are presented as mean (standard deviation [SD] and/or as median (inter-quartile range [IQR]). All data were analyzed using Microsoft Excel and conducted under Windows XP Professional.

## Results

A total of 75,157 admissions (day case or inpatient) for Palmar Fascial Fibromatosis (ICD10 = M720) as either a primary or subsidiary diagnosis were recorded in England between April 2003 and March 2008. Of these, 71,103 admissions (94.6%) were considered for further analysis (4,054 admissions were excluded due to unrelated patient death, no recorded procedure or trauma). The records were more closely reviewed, and an additional 6,597 patients were excluded because there was no reference to a procedure to correct Dupuytren's contracture having been conducted at that admission.

**Figure 1:**
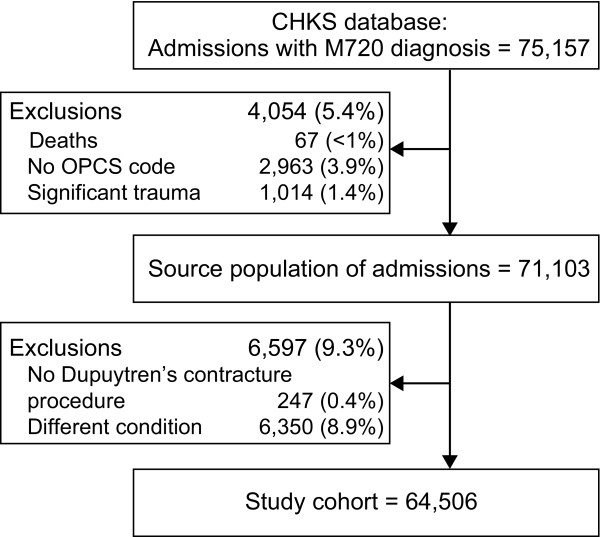
**Patient flow**.

The current analyses are based upon 64,506 evaluable hospital admissions recorded during the 5-year period (Figure 1). Of this total, 51,284 (80%) were male patients (63 ± 11 y), and 13,220 (20%) were female patients (66 ± 11 y). These figures varied little over time. The number of admissions per year was 12,901 ± 330 and also remained stable.

**Figure 2:**
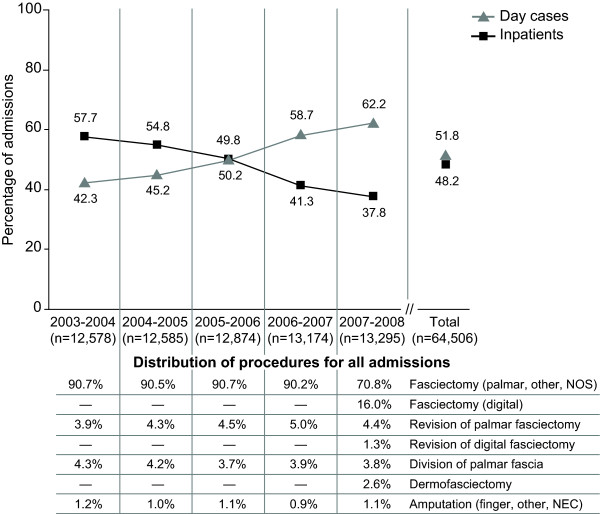
**Day-case and inpatient admission rates by year and type of Dupuytren's procedure**.

Overall, day-case admission rates increased from 42% in 2003-2004 to 62% in 2007-2008, and inpatient admission rates decreased from 58% to 38%, respectively (Figure 2). Between April 2003 and March 2007, Fasciectomy (Palmar [T521], Other specified excision [T528] and Unspecified excision [T529]) accounted for 91% of elective procedures performed. Revision of Palmar Fasciectomy (T522) and Division of Palmar Fascia (T541) each accounted for ~4% of procedures; Amputations (X083, X084, X088) accounted for the remaining 1%. Between April 2007 and March 2008, Fasciectomy rates decreased to 71%, owing to the introduction of codes for Digital Fasciectomy (T525), its Revision (T526) and Dermofasciectomy (T561), which together accounted for 20% of procedures that year. Rates for the other procedures remained unchanged. There were no differences in procedure rates by year between day-case (Table 1) and inpatient admissions (Table 2).

**Table 1:** Summary of Dupuytren's contracture procedures for day-case admissions by OPCS code and year

OPCS	Description	2003-2004		2004-2005		2005-2006		2006-2007		2007-2008	
**Code**	***Fasciectomy***	**n**	**%**	**n**	**%**	**n**	**%**	**n**	**%**	**n**	**%**

T521	Palmar Fasciectomy	4,722	88.8	5,021	88.2	5,701	88.9	6,736	87.2	5,888	71.2
T525	Digital Fasciectomy	--	--	--	--	--	--	--	--	1307	15.8
T528	Other specified excision of other fascia	39	0.7	50	0.9	79	1.2	146	1.9	30	0.4
T529	Unspecified excision of other fascia	63	1.2	89	1.6	90	1.4	130	1.7	54	0.7
	*Total*	*4824*	*90.7*	*5160*	*90.7*	*5870*	*91.5*	*7012*	*90.7*	*7279*	*88.0*

	***Revision of Fasciectomy***										
T522	Revision of Palmar Fasciectomy	176	3.3	189	3.3	213	3.3	320	4.1	322	3.9
T526	Revision of Digital Fasciectomy	--	--	--	--	--	--	--	--	90	1.1
	*Total*	*176*	*3.3*	*189*	*3.3*	*213*	*3.3*	*320*	*4.1*	*412*	*5.0*

	***Fasciotomy***										
*T541*	*Division of Palmar Fascia*	*264*	*5.0*	*298*	*5.2*	*272*	*4.2*	*330*	*4.3*	*352*	*4.3*

	***Dermofasciectomy***										
*T561*	*Dermofasciectomy*	*--*	*--*	*--*	*--*	*--*	*--*	*--*	*--*	*134*	*1.6*

	***Amputations***										
X083	Amputation of phalanx of finger	10	0.2	7	0.1	18	0.3	20	0.3	28	0.3
X084	Amputation of finger NEC	43	0.8	35	0.6	38	0.6	45	0.6	64	0.8
X088	Other specified amputation of hand	2	0.0	1	0.0	1	0.0	1	0	0	0.0
	*Total*	*55*	*1.0*	*43*	*0.8*	*57*	*0.9*	*66*	*0.9*	*92*	*1.1*

	**Grand Total**	**5,319**		**5,690**		**6,412**		**7,728**		**8,269**	

**Table 2:** Summary of Dupuytren's contracture procedures for inpatient admissions by OPCS code and year

OPCS		2003-2004		2004-2005		2005-2006		2006-2007		2007-2008	
**Code**	***Fasciectomy***	**n**	**%**	**n**	**%**	**n**	**%**	**n**	**%**	**n**	**%**

T521	Palmar Fasciectomy	6,355	87.5	5,975	86.7	5,661	87.6	4,706	86.4	3,352	66.7
T525	Digital Fasciectomy	--	--	--	--	--	--	--	--	819	16.3
T528	Other specified excision of other fascia	168	2.3	182	2.6	97	1.5	113	2.1	43	0.9
T529	Unspecified excision of other fascia	56	0.8	78	1.1	52	0.8	47	0.9	42	0.8
	*Total*	*6,579*	*90.6*	*6235*	*90.4*	*5,810*	*89.9*	*4,866*	*89.3*	*4,256*	*84.7*

	***Revision of Fasciectomy***										
T522	Revision of Palmar Fasciectomy	309	4.3	347	5.0	370	5.7	344	6.3	266	5.3
T526	Revision of Digital Fasciectomy	--	--	--	--	--	--	--	--	80	1.6
	*Total*	*309*	*4.3*	*347*	*5.0*	*370*	*5.7*	*344*	*6.3*	*346*	*6.9*

	***Fasciotomy***										
*T541*	*Division of Palmar Fascia*	*279*	*3.8*	*232*	*3.4*	*198*	*3.1*	*180*	*3.3*	*154*	*3.1*

	***Dermofasciectomy***										
*T561*	*Dermofasciectomy*	*--*	*--*	*--*	*--*	*--*	*--*	*--*	*--*	*210*	*4.2*

	***Amputations***										
X083	Amputation of phalanx of finger	24	0.3	21	0.3	20	0.3	11	0.2	11	0.2
X084	Amputation of finger NEC	66	0.9	59	0.9	61	0.9	45	0.8	48	1.0
X088	Other specified amputation of hand	2	0.0	1	0.0	3	0.0		0.0	1	0.0
	*Total*	*92*	*1.3*	*81*	*1.2*	*84*	*1.3*	*56*	*1.0*	*60*	*1.2*

	**Grand Total**	**7,259**		**6,895**		**6,462**		**5,446**		**5,026**	

Overall, 79% of admissions were overseen by trauma and orthopaedic surgeons and 19% by plastic surgeons; these proportions varied by less than ±1% in each 12-month period. Few admissions were overseen by physicians outside these specialties: about 1% (n = 612) were performed by emergency physicians and <0.5% (n = 181) by general surgeons over the 5-year period (Table 3). Although the most commonly performed procedure across surgical specialties was fasciectomy, orthopaedic and plastic surgeons performed a wider variety of procedures.

**Table 3:** Procedures for Dupuytren's contracture by physician specialty and OPCS code

OPCS		Trauma & Orthopaedic		Plastic Surgery		Accident & Emergency		General Surgery	
**Code**	**Description**	**n**	**%**	**n**	**%**	**n**	**%**	**n**	**%**

T521	Palmar Fasciectomy	42,959	66.6	10,401	16.1	536	0.8	166	0.3
T525	Digital Fasciectomy	1,579	2.4	531	0.8	14	0.0	1	0.0
T528	Other specified excision of other fascia	741	1.1	202	0.3	0	0.0	4	0.0
T529	Unspecified excision of other fascia	565	0.9	130	0.2	4	0.0	0	0.0
T522	Revision of Palmar Fasciectomy	2,250	3.5	568	0.9	37	0.1	0	0.0
T526	Revision of Digital Fasciectomy	148	0.2	22	0.0	0	0.0	0	0.0
T541	Division of Palmar Fascia	2,188	3.4	334	0.5	16	0.0	9	0.0
T561	Dermofasciectomy	223	0.3	121	0.2	0	0.0	0	0.0
X083	Amputation of phalanx of finger	124	0.2	46	0.1	0	0.0	0	0.0
X084	Amputation of finger NEC	412	0.6	86	0.1	5	0.0	1	0.0
X088	Other specified amputation of hand	9	0.0	3	0.0	0	0.0	0	0.0

	**Total**	**51,198**	**79.4**	**12,444**	**19.3**	**612**	**0.9**	**181**	**0.3**

**Figure 3:**
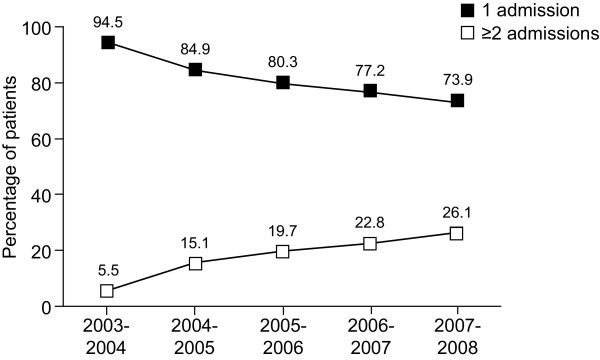
**Number of admissions per patient per year for a Dupuytren's procedure**.

Between April 2003 and March 2004, 7,259 admissions received a Dupuytren's contracture procedure that was recorded as having been performed on an inpatient basis, which was defined a priori as ≥1-night stay in hospital. However, for 13% (n = 910) of these admissions, patients were discharged on the same day of the procedure. In fact, these percentages rose steadily thereafter, up to 26% in 2008. Within each 12-month period, the largest percentage of miscoded inpatient admissions was for Amputations, although the overall frequency of these procedures was relatively low. For the majority of procedures performed in 2007-2008, more than 25% were miscoded as inpatient admissions. Length of stay decreased slightly from 1.48 ± 1.4 days in 2003-2004 to 1.03 ± 1.2 days in 2007-2008; however, variation from year to year and across procedures was quite large (Table 4). Lastly, the number of admissions per patient per year for a Dupuytren's procedure increased steadily between 2003 and 2008. In 2008, 26% of patients had two or more admissions for a Dupuytren's procedure (Figure 3).

**Table 4:** Length of stay for inpatient admissions by year and OPCS code

OPCS		2003-2004		2004-2005		2005-2006		2006-2007		2007-2008	
**Code**	**Description**	**Mean**	**SD**	**Mean**	**SD**	**Mean**	**SD**	**Mean**	**SD**	**Mean**	**SD**

T521	Palmar Fasciectomy	1.48	1.34	1.35	2.56	1.29	1.42	1.17	1.61	1.04	1.31
T525	Digital Fasciectomy	--	--	--	--	--	--	--	--	0.97	1.04
T528	Other specified excision of other fascia	1.60	1.08	1.48	0.81	1.16	1.04	1.05	0.95	0.98	0.94
T529	Unspecified excision of other fascia	1.30	1.01	1.37	0.97	1.08	0.84	0.87	0.68	1.31	2.09
T522	Revision of Palmar Fasciectomy	1.54	1.95	1.31	1.09	1.28	1.07	1.23	0.96	1.10	1.02
T526	Revision of Digital Fasciectomy	--	--	--	--	--	--	--	--	0.89	0.75
T541	Division of Palmar Fascia	1.57	2.47	1.14	1.17	1.16	1.25	1.44	3.48	0.95	0.92
T561	Dermofasciectomy	--	--	--	--	--	--	--	--	1.14	0.76
X083	Amputation of phalanx of finger	1.38	1.10	1.05	0.80	1.10	1.09	1.18	0.98	0.91	0.70
X084	Amputation of finger NEC	1.32	1.45	1.41	1.10	1.07	1.30	1.07	0.78	1.02	0.96
X088	Other specified amputation of hand	2.50	0.71	1.00	0	1.00	0	0	0	0	0

	**Overall**	**1.48**	**1.42**	**1.35**	**2.41**	**1.28**	**1.39**	**1.17**	**1.65**	**1.03**	**1.22**

Per-patient and total estimated costs for 2010-2011 varied by procedure type and patient status (Table 5). One-year total costs for DC were £41,576,141. Day case and inpatient admissions accounted for 57% (£23,834,242) and 43% (£17,741,900) of total costs, respectively. Overall, mean per-patient costs were £2,885 (day case) and £3,534 (inpatient). Per-patient costs for Palmar Fasciectomy were £2,736 for day cases and £2,785 for inpatients; respective costs for Digital Fasciectomy were £3,148 and £5,142. Per-patient costs for Palmar Revision were £2,794 for day cases and £4,332 for inpatients; respective costs for Digital Revision were £9,210 and £9,208. The majority of costs (82%; £33,938,028) were for fasciectomy procedures. Revisions of a fasciectomy accounted for 9% (£3,617,572) of total costs (Table 6).

**Table 5:** Summary of 1-year (2010-2011) total and per-patient costs by OPCS code, HRG code and admission type

		Procedures	HRG	Mean Total Costs (£)			Mean Per-Patient Costs (£)		
		
OPCS	Description	n (%)	Code	Day Case	Inpatient	All	Day Case	Inpatient	All
T521	Palmar Fasciectomy	9,240 (69.5)	HB53Z	15,778,965	8,856,210	24,365,176			
			HB52C	213,008	257,761	470,769			
			HB52B	94,066	206,328	3,000,394			
			**Total**	**16,086,039**	**9,320,300**	**25,406,338**	**2,736**	**2,785**	**2,750**

T525	Digital Fasciectomy	2,126 (16.0)	HB51Z	3,976,776	4,128,570	8,105,346			
			HA06Z	138,075	82,845	220,920			
			**Total**	**4,114,851**	**4,211,415**	**8,326,266**	**3,148**	**5,142**	**3,916**

T528	Other specified excision of other fascia	73 (0.5)	HB55C	26,180	30,940	5,7120			
			HB55B	8,806	18,870	27,676			
			HB54C	1,880	3,760	5,640			
			**Total**	**36,866**	**53,570**	**90,436**	**1,229**	**1,246**	**1,239**

T529	Unspecified excision of other fascia	96 (0.7)	HB55C	57,120	44,030	101,150			
			HB55B	7,548	6,290	13,838			
			**Total**	**64,668**	**50,320**	**114,988**	**1,198**	**1,198**	**1,198**

T522	Revision of Palmar Fasciectomy	588 (4.4)	HB52C	645,460	581,803	1,227,263			
			HB52B	229,632	432,960	662,592			
			HB51Z	24,624	137,619	162,243			
			**Total**	**899,716**	**1,152,382**	**2,052,098**	**2,794**	**4,332**	**3,490**

T526	Revision of Digital Fasciectomy	170 (1.3)	HA06Z	810,040	727,195	1,537,235			
			HA05Z	18,826	9,413	28,239			
			**Total**	**828,866**	**736,608**	**1,565,474**	**9,210**	**9,208**	**9,209**

T541	Division of Palmar Fascia	506 (3.8)	HB55C	320,110	94,010	414,120			
			HB55B	88,060	81,770	169,830			
			HB54C	15,040	3,760	18,800			
			HB54B	10,860	17,376	28,236			
			**Total**	**434,070**	**196,916**	**630,986**	**1,233**	**1,279**	**1,247**

			HA06Z	1,205,855	1,914,640	3,120,495			
T561	Dermofasciectomy	344 (2.6)	HA05Z	28,239	18,826	47,065			
			**Total**	**1,234,094**	**1,933,466**	**3,167,560**	**9,210**	**9,207**	**9,208**

X083	Amputation of phalanx of finger		HB55C	55,930	29,750	85,680			
			HB54C	43,240	13,160	56,400			
X084	Amputation of finger NEC	152 (1.1)	HB55B	16,354	23,902	40,256			
			HB54B	19,548	17,376	36,924			
X088	Other specified amputation of hand		HB53Z	0	2,735	2,735			
			**Total**	**135,072**	**86,923**	**221,995**	**1,468**	**1,449**	**1,460**

	**Overall total costs in England**			**23,834,242**	**17,741,900**	**41,576,141**	**2,885**	**3,534**	**3,127**

**Table 6:** Summary of 1-year (2010-2011) total costs by OPCS code, admission type and percentage of total costs

		Day Case		Inpatient		All Admissions	
		
OPCS	Description	Costs (£)	%	Costs (£)	%	Costs (£)	%
T521	Palmar Fasciectomy	16,086,039	67.5	9,320,300	52.5	25,406,338	61.1
T525	Digital Fasciectomy	4,114,851	17.3	4,211,415	23.7	8,326,266	20.0
T528	Other specified excision of other fascia	36,866	0.2	53,570	0.3	90,436	0.2
T529	Unspecified excision of other fascia	64,668	0.3	50,320	0.3	114,988	0.3
	
	Fasciectomy total	20,302,424	85.2	13,635,605	76.9	33,938,028	81.6
							
T522	Revision of Palmar Fasciectomy	899,716	3.8	1,152,382	6.5	2,052,098	4.9
T526	Revision of Digital Fasciectomy	828,866	3.5	736,608	4.2	1,565,474	3.8
	
	Revision total	1,728,582	7.3	1,888,990	10.6	3,617,572	8.7
							
T541	Fasciotomy	434,070	1.8	196,916	1.1	630,986	1.5
							
T561	Dermofasciectomy	1,234,094	5.2	1,933,466	10.9	3,167,560	7.6
							
X083, X084, X088	Amputation	135,072	0.6	86,923	0.5	221,995	0.5

## Discussion

We extracted and reviewed HES data to evaluate trends in the clinical management of Dupuytren's disease in England during the 5-year period from April 2003 through March 2008 and to estimate 1-year NHS costs for 2010-2011. Specifically, we systematically summarized information about the total number of hospital admissions and the average length of stay, the types of surgical procedures used and the frequency with which they were performed and by whom, and whether these procedures were performed on a day-case or inpatient basis.

Overall, the mean number of admissions remained fairly stable from year to year, and patient demographics such as mean age and gender distribution varied little over time. Similarly, in a 10-year review of data from the Pulvertaft Hand Centre, there was little change in the age of presenting patients between 1990 and 2000 [8]. Between 2003 and 2007, the vast majority (91%) of elective procedures were coded as Fasciectomy (Palmar, Other, Unspecified); the remaining procedures were coded as Revision of Fasciectomy (4%), Fasciotomy (4%) and Amputation (1%). Procedure rates were also stable during this time, and rates for day cases versus inpatients were comparable. Between 2007 and 2008, traditional Fasciectomy rates declined to 71%, owing to the introduction of OPCS codes for Digital Fasciectomy, its Revision and Dermofasciectomy, which accounted for 16%, 1.3% and 2.6% of procedures, respectively, during this 12-month period. One-year total costs for DC were £41,576,141 with day cases accounting for 57% (£23,834,242) of total costs. Mean per-patient costs were £2,885 (day case) and £3,534 (inpatient). Because costs associated with follow-up visits and physiotherapy were not included in the analysis, the values are highly likely to be underestimates of the total direct costs of the management of Dupuytren's disease in England.

Few studies have assessed the treatment costs on a national level for Dupuytren's disease. To our knowledge, our cost analysis was the first conducted for England for Dupuytren's disease. Using the French National Database, Maravic and Landais conducted a similar study to evaluate elective admissions of men and women (aged ≥46 y) with a primary ICD-10 diagnosis code (ie, M72.0, M72.00, M72.04, M27.09) and surgical code for Dupuytren's disease in 2001. In total, 14,860 cases were identified. Four procedures were described: needle fasciotomy, surgical fasciectomy of one finger, surgical fasciectomy of two or more fingers, and re-intervention [9]. Overall, a Dupuytren's procedure occurred mostly in men (82%), was managed primarily in private hospitals (77%) with a short length of stay (≤24 h; 53%), and fasciectomy was the most common surgical procedure (88%). Total hospital costs (2005) for all elective admission was €14,179,998 [9]. In a retrospective analysis of hospital admissions spanning 50 years, Loos et al [10] showed that between 1988 and 2006, 95% (1,061/1,119) of operated Dupuytren's disease patients had a limited fasciectomy, 5% (58/1,119) had total fasciectomy, and 1% (13/1,119) had amputations. Of this total, 12% of surgeries were for recurrent contracture [10].

For each year of the 5-year period, we show that the majority of Dupuytren's contracture procedures were performed by trauma and orthopaedic surgeons (79%) followed by plastic surgeons (19%); a small percentage of procedures were performed by emergency physicians or general surgeons. In a similar analysis of HES data for 2001, Hobby and Dias showed that three main surgical specialties treated hand conditions: 67% were orthopaedic surgeons and 31% were plastic surgeons. A small percentage (1%) was represented by trauma surgeons [11]. In a 6-month study of 76 consecutive Danish patients with advanced Dupuytren's disease, day case surgery was performed on 96% (50/52) of patients with primary contracture and 79% (19/24) of those with recurrent disease [12]. Senior registrars from the Department of Orthopaedic Surgery performed 89% of primary surgeries and 50% of recurrent surgeries. The remaining 50% of recurrent surgeries were performed by a specialist in hand surgery. There were no differences between the primary and recurrent group in terms of complications; however, 10% of primary cases had perioperative nerve injuries [12]. Both studies concluded that all surgeries for Dupuytren's disease should be performed by experienced surgeons with proper training in hand surgery.

While our findings show minimal changes in the number of admissions per year, patient demographics, procedure rates, and physician specialty between April 2003 and March 2008, there was a clear reversal in the distribution of admissions treated as day cases versus inpatients. In 2000, the Department of Health's NHS Plan recommended that 75% of elective admissions should be day cases [13], and excision of Dupuytren's contracture is among the 25 'basket' procedures that should normally be performed as such [14]. In one report, the percentage of day-case versus inpatient procedures for Dupuytren's contracture was 30% in 1996-1997; by 2003-2004, the rate had increased to 43% [15]. In our study, day-case admission rates increased from 42% in 2003-2004 to 62% in 2007-2008. These results are also comparable to those reported by The Health Commission in their latest review of HES data: day-case rates for Dupuytren's contracture excision rose gradually from ~35% in 1998-1999 to ~40% in 2003-2004 [14]. In the Healthcare Commission report, day-case procedure rates for Dupuytren's contracture were classified as 'still rising' at a medium-to-high rate [14], which is consistent with our findings for 2007-2008. Interestingly, between April 2007 and March 2008, while the number of admissions recorded as inpatients fell by 31%, the proportion that did not have an overnight stay doubled to 26% (n = 1295). Indeed, there was a steady increase in the number of admissions coded as inpatient (requiring ≥1-night stay in hospital), but where the patient was discharged the same day. When a patient did have a hospital stay, the mean duration was 1.5 days in 2003-2004 and 1 day in 2007-2008.

The review of HES data suggests that the number of admissions per patient per year for a Dupuytren's procedure increased steadily between 2003 and 2008. One possible explanation is an increase in the staging of corrective procedures by surgeons who are more concerned about achieving positive outcomes. By contrast, it may be that surgeons are rushing procedures owing to diminished resources that may include a shortage of qualified surgeons and/or decreased operating theatre time. This may result in an increased incidence of infection, graft failure or other complications that require additional admission. A third option is that as waiting lists decrease, patients with multiple affected joints in the same hand or bilateral contractures can receive more than one procedure in a single calendar year.

Historically and relative to other Western countries, the UK has been slow to adopt a day-case approach to surgery for Dupuytren's disease [16]. In the early 1990s, a survey of 24 hand surgeons showed that 25% never carried out Dupuytren's surgery on a day-case basis; the remaining surgeons did so occasionally. In no case was day-case surgery part of their routine practice; 71% admitted patients for ≥2 nights [16]. In a follow-up study to investigate the effectiveness of regional anaesthesia and the safety of surgery performed on a day-case basis, the investigators used brachial plexus block and local fasciectomy in 50 Dupuytren's disease patients. No patient required general anaesthesia and only one patient who experienced a hypotensive episode during surgery was admitted overnight. All other patients were discharged later the same day [16]. Similarly, during a 6-month study conducted at the Pulvertaft Hand Centre, 1,003 patients presented for elective hand surgery. Between 1990 and 2000, the referral rate for elective surgeries had increased by 36%, from 289 to 392 per 100,000 population per year. The day-case surgery rate increased from 64% to 94%, and the number of inpatient days decreased from 221 to 210 per 100,000 population per year [17]. Dupuytren's disease was the fifth most common elective surgery performed in the clinic at 33 per 100,000 population per year. Thus, Dupuytren's disease accounted for 8% of the elective hand surgeries during the time period examined.

These findings should be considered in light of some limitations. First, although care is taken to avoid inaccuracies in the HES database - and CHKS audits more than 60% of records - there will be errors and omissions that are not reviewed and/or corrected on an ongoing basis. It is reasonable to assume, however, that such inaccuracies are random occurrences and no systematic biases are introduced. As a census-based study, there are no ethnic or geographic biases in the data; they are representative of the overall population in England that receives hospital-based care for Dupuytren's contracture. Nevertheless, the database does not include information about procedures conducted as an outpatient or from patients treated in the private sector, so results cannot be generalized to these subpopulations in England.

## Conclusions

Between 2003 and 2008, fasciectomy was the most common and most costly surgical procedure for Dupuytren's contracture in England. While procedure rates and physician specialties varied little during this time, there was a marked change in patient status and overnight stays: inpatient operations decreased as day-case procedures increased. The change was likely due to economic trends and modifications to the healthcare system in England. Future studies are warranted to monitor these trends and the associated effects on outcomes and costs.

## Competing interests

RAG and RT are employees of Pfizer Inc and Pfizer Ltd, respectively. RP is an employee of Adelphi who serve as consultants to Pfizer Inc and were funded by Pfizer for data analysis and manuscript development. CB also serves as a consultant to Pfizer.

## Authors' contributions

RAG, RP and CB proposed key elements for and made significant contributions to the study design and analysis; RP was the liaison with Caspe Healthcare Knowledge Systems (CHKS); RAG and RP developed the appropriate methodology for the analysis and played a key role in the evaluation and assessment of the results. RT and CB participated in the evaluation of the data, placing the results within the context of the current literature; CB assisted in placing the results in the context of clinical practice. All authors provided direction and intellectual content for the manuscript, participated in reviews and submitted written approval of the final version.

## Pre-publication history

The pre-publication history for this paper can be accessed here:

http://www.biomedcentral.com/1471-2474/12/73/prepub
